# Recent advances of integrated microfluidic suspension cell culture system

**DOI:** 10.1049/enb2.12015

**Published:** 2021-10-11

**Authors:** Yi Jing Kerk, Aysha Jameel, Xin‐Hui Xing, Chong Zhang

**Affiliations:** ^1^ Institute of Biochemical Engineering Department of Chemical Engineering, Tsinghua University Beijing China; ^2^ MOE Key Laboratory of Industrial Biocatalysis Department of Chemical Engineering, Tsinghua University Beijing China; ^3^ Center for Synthetic and Systems Biology Tsinghua University Beijing China

**Keywords:** bioprocess engineering, microbial synthesis, optimisation, synthetic biology, screening strategies, bioMEMS, cellular biophysics, drops, drugs, lab‐on‐a‐chip, microfluidics, suspensions, microfabrication

## Abstract

Microfluidic devices with superior microscale fluid manipulation ability and large integration flexibility offer great advantages of high throughput, parallelisation and multifunctional automation. Such features have been extensively utilised to facilitate cell culture processes such as cell capturing and culturing under controllable and monitored conditions for cell‐based assays. Incorporating functional components and microfabricated configurations offered different levels of fluid control and cell manipulation strategies to meet diverse culture demands. This review will discuss the advances of single‐phase flow and droplet‐based integrated microfluidic suspension cell culture systems and their applications for accelerated bioprocess development, high‐throughput cell selection, drug screening and scientific research to insight cell biology. Challenges and future prospects for this dynamically developing field are also highlighted.

## 1 INTRODUCTION

Cell culture is a versatile tool to improve our understanding of cell biology, morphology and physiology, and also an essential process for cell clonal expansion, and acquisition of various biological products. In general, cell culture has been performed in suitable media‐contained traditional lab apparatus (e.g. petri dishes, microtiter plates, shake flasks, and bioreactors) to preserve the bulk population of cells alive. Over the years, with developments of in‐depth study in cell biology‐related fields, there is an increasing demand to elucidate complex cellular events and profile cell dynamics quantitatively [1, 2, 3]. Since this research has been exasperated due to variations among individual cells that cannot be detected under bulk cell population in conventional culture experiments, effective cell culture strategies that highlighted phenotypic heterogeneity have been gaining more interest [4, 5]. Moreover, development in biotechnology and bioproduction requires abundant experiments to optimise culture parameters and strategies, which has been a labour‐intensive and expensive process [6]. The advent of microfluidic technology as a promising cell culture platform has overcome these challenges. Microfluidic technology allows to manipulate fluidics in a micrometre‐scale channel that is compatible with the cell size. It showed huge potential in cell research by offering the following advantages [3, 7] (i) Miniaturisation of working volume increases the sensitivity and culture efficiency; (ii) High‐throughput manner enhances the parallelisation and data acquisition in a single experiment; (iii) Precise and automated fluid control allows accurate manipulation in different culture conditions; (iv) Low reagent or sample consumption offers cost‐effective operations; (v) Modular design, flexible schedule and strong adaptability allow for multiple tasks; (vi) Ease of fabrication to meet different culture demands; (v) The microfluidics platform can promise more physiologically relevant microenvironments using bio‐compatible materials (PDMS, glass, silicon) to make it a cell‐friendly environment that can be easily tunable according to user's needs [8].

**FIGURE 1:**
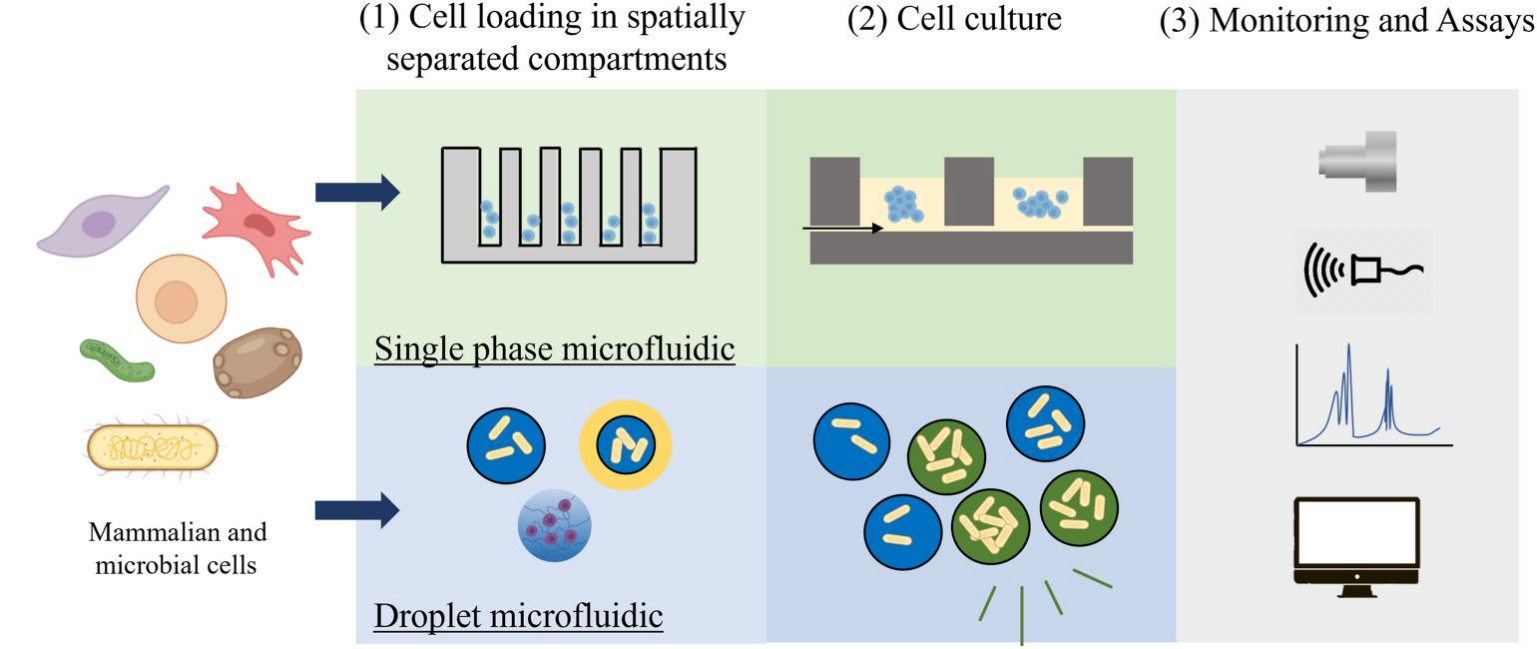
Illustration of microfluidic cell culture systems adapting the basic culture protocol, includes (a) cell loading in a specific region, (b) culturing and incubating the cell, and (c) monitoring key parameters and performing cell‐based assays on demand

Nowadays, various custom‐designed microfluidic substrates with uptrend integration of auxiliary functional components (e.g. microchambers, micropumps, microvalves, sensors, microscopies etc.) have offered simple and flexible culture protocols to realize the lab‐on‐a‐chip concept. Generally, a versatile microfluidic cell culture system could perform the following steps in a single platform: (1) cell loading and patterning in a specified region, (2) cell culture in a defined and monitored condition, and (3) cell manipulation for screening, sorting, phenotype selection and various assays, as illustrated by Figure 1. The system integrity has particularly contributed to realize a continuous, stable, controllable and programmable culture process. Moreover, the capability to carry out massive parallel and sequential operations also enhanced practicality [1], for example culturing the cell in numerous chambers simultaneously and further phenotype‐based screening. These unique features have paved the way for novel methods to advance cell interaction networks [9], cell programming [10], cell‐based drug screening [11, 12] and exploring biological diversity [13].

In this review, we focus on the recent advances and applications of integrated and functionally equipped microfluidic culture systems, in particular for suspension culture, that is mammalian and microbial cells. Subsequently, we discuss the multitude of challenges and future perspectives of integrated microfluidic cell culture systems.

## 2 INTEGRATED MICROFLUIDIC CELL CULTURE SYSTEM

As microfluidic cell culture systems have been positioned as a powerful cell culture platform for biotechnological, biomedical and bioscience research, two major types of the advanced microfluidic approach have been developed, which are single‐phase and droplet‐based cell culture systems. The major characteristics and functions have been summarised in Table 1. The key idea of integrated bioprocess is to reduce time consumption, footprints and consequently a streamlined workflow. Hence, the important considerations for automated workflow operation include the following: (1) the geometry and dimensions of the culture region that can determine experimental throughput, and (2) the integration of functional elements to contribute for automation and functionality on fluid control and parameter monitoring for further analysis.

**TABLE 1:** Major characteristics and integrated functions of the microfluidic cell culture system

Microfluidic types	Culture form	Working volume	Parallelisation	Monitoring parameters	Function	Application
**Single‐phase flow culture**	**Well**	1∼100 nL [14, 15]	<10 wells [20]	Fluorescence intensity [14, 15, 16, 20]	Feedback control [19]	Process development [18, 19, 20]
		1∼100 μL [16, 17]	16 wells [16, 19]	OD [18, 19]	Data detection [18, 19]	Cell selection [14, 19]
		0.1∼1 ml [18]	32 wells [17, 18]	pH [18]	Image acquisition [14, 15, 16, 17]	Long‐term culture [15, 17, 19]
		20∼40 ml [19]	1600 chambers [15]		Flow control [15, 16, 17, 18, 19, 20, 21]	Drug test [16]
			2172∼2832 microwells [14]		pH control [18]	
					Temperature control [19]	
	**Channel/chamber**	0.1∼1 nL [22]	<10 chambers [23, 24, 26, 27, 28]	Fluorescence intensity [22, 23, 25, 26, 29, 30]	Feedback control [22, 27]	Process development [21]
		1∼100 nL [23]	12 units [21]	OD [21, 27]	Data detection [21, 22, 27]	Kinetic parameter determination [24]
		0.1∼1 μL [24, 25]	24 chambers [25]	DO [21, 27]	Image acquisition [22, 23, 24, 25, 28, 29, 30]	Long‐term culture [22, 23, 27, 28]
		1∼100 μL [21, 26]	32 chambers [29]	pH [27]	Flow control [21, 25, 26, 27, 30, 31]	Drug test [25, 26, 29]
		0.1∼1 ml [27]	150 chambers [30]	Impedimetric analysis [26, 28]		3D cell culture [26]
			500 chambers [22]			Co‐culture [30]
**Droplet‐based culture**	**Water/Oil**	0.01∼1 nL [29, 32, 35, 36, 37, 38, 39, 40, 42]	Droplets Amount:	Fluorescence intensity [32, 33, 35, 36, 38, 39, 41]	Data detection [32, 33, 34, 35, 37, 38]	Cell selection [32, 33, 35, 42]
	W/O [29, 32, 33, 34, 35, 36, 37, 38, 39, 40]	1∼100 nL [33]	≤1000 droplets [34, 39]	OD [34, 37]	Sorting [32, 33, 34, 36, 37, 42]	Drug test [29, 37, 38, 39, 40]
	W/O/W [41]	2 μL [34]	10^3^∼10^5^ [29, 35, 38, 40, 42]	Bright field Cell growth [40]	Image acquisition [29, 35, 36, 37, 38, 39, 40, 41, 42]	Biodiversity study [29, 36, 37]
			10^5^∼10^7^ [32, 36, 37]		Splitting, Fusion [34]	Adaptive evolution [34]
			Generating frequency:		Pico‐injection [35, 36, 42]	
			20∼100 Hz [33]		Streaking [29]	
			2800 Hz [32]			
	**Multi‐aqueous**	<50 pL [43, 44]	Droplets Amount:	Fluorescence intensity [43, 44, 45, 47]	Sorting [43, 44]	Cell selection screening [43, 44]
	Hydrogel [43, 44, 45]	0.1∼1 nL [46]	10^2^ microcapsules [47]		Oil filtering and Crosslink [45]	3D cell culture [43, 45, 47]
	W/W [46, 47]	1∼100 nL [45, 47]	>10^3^ microparticles [44]		Image acquisition [43, 45, 47]	
			Generating frequency:			
			100 Hz [46]			
			1200 Hz [43, 44]			

Compartmentalisation is an excellent approach to increase process efficiency and analysis sensitivity [48], which allows individual operation without affecting others. It is feasible to adopt a wide range of parallel compartments (from 10^0^ to 10^3^) and working volumes (from ml to nL) of culture events in a single‐phase culture platform. In contrast, the droplet‐based culture system demonstrated an outstanding partitioning capacity (typically pL to μL) and throughput (for up to 10^7^) for enhanced culture and screening efficiency. Cell studies on the physiological behaviour and morphological characteristics at different levels of resolution can be carried out by changing the culture volume, for example pL and nL scale culture compartments can be used to emphasize cellular heterogeneity at the single cell level whereas the nL, μL and mL culture systems are suitable for whole cell community‐based biological assays. Miniaturised culture volumes featured a homogenous environment and high surface‐to‐volume ratio for enhanced mass transfer [49]. Furthermore, improved experimental throughput associated with compartmentalisation and parallelisation would be a critical indicator to evaluate the microfluidic cell culture performance.

Other versatile features of microfluidic systems include its incorporated functional fluid control components (externally and internally) and detection techniques to facilitate the culture process. In most situations, cell manipulation coupled with sophisticated fluid control, such as cell seeding in a specific position used to regulate cell growth under dynamic culture conditions and the transport or sampling of cultured cells. For example, pump‐valves integration has been applied in PDMS‐based chip to guide fluid flow [25], addressable cell extraction, loading, controlled cells contact [50, 51] and encapsulating cells within droplets [48], reagent perfusion [10] and other derived functions such as medium circulation for controlled shear stress [52]. Moreover, online monitoring of the culture process is also important to quantitatively characterise the dynamic changes in terms of growth condition, morphological changes, physiological responses and metabolites secretion. This information is valuable to determine the cell‐growth conditions and underlying biological mechanisms coupled to subtle alternation of culture conditions. Therefore, integration of sensors (optical, electrochemical [53, 54, 55], biosensors [56] etc.) and microscopic technologies enabled automatic collection of measurable culture data, for example pH, optical density (OD), dissolved oxygen (DO), nutrients intake, phenotyping fluorescent intensity as well as cells’ appearance. Certain sensing techniques subsequently applied for automated feedback culturing, analysis and sorting process, for example chemostat continuous culture [57], phenotypic screening, protein screening [53, 58], and immunoassays [59]. The integration of these analytical detection technologies in a single culture system improved the data acquiring performance by reducing analysis time and increasing reliability through automation. It also indicates that more operations can be programmed in one platform, and eventually the operational error caused by human interference or potential exposure to undesired contamination when transporting cell samples for subsequent analysis can also be minimised.

Suspension mammalian and microbial cell culture techniques have been generally used in the biotechnological production and laboratorial studies. Due to the intrinsic characteristics of mammalian cell culture, it usually requires a scaffold or surface to support their growth, and it is highly sensitive to culture conditions [60], that is pH, extracellular matrix (ECM) heterogeneity, temperature, flow shear stress, aseptic condition and many others. The advent of microfluidics enhanced suspension culture performance by providing shear‐free or shear‐stress controlled conditions [61, 62], encapsulate the cells in hydrogel beads to create 3D in vivo physiologically alike scaffold for adherent conditions [63] as well as carry out the dynamic perfusion and static culture [64]. For example, the incorporation of microfluidics with the hanging droplet technique allowed the formation, maintenance and drug resistance assessment of multicellular spheroids [65]. The microfluidic chips were placed in incubators for cell proliferation under controlled CO_2_ concentration, temperature and humidity environment [66], while the gas permeable microfluidic materials were used to ensure gas exchange in culture medium [67]. It also contributed for the improvement of microbial suspension culture, while microfluidics also provide oxygen supply and additional nutrients for extended time period (usually in days) to the cell culture platform [23, 68]. The metabolic mechanisms of microorganisms can be discovered by encapsulating few cells in droplets which allow microbial consortia form within confinement and avoid its potential contamination from air during agar plate incubation. Droplets‐based microfluidics also helps to prevent the cross‐contamination and biofilm formation during long‐term bacteria culture, usually by enforcing continuous movement of droplets to avoid the physical contact with microfluidic channels and walls [69]. The innovation of microfluidics will be further discussed with detailed examples in the following sections.

### 2.1 Single‐phase flow: well‐based and channel/chamber‐based culture

**FIGURE 2:**
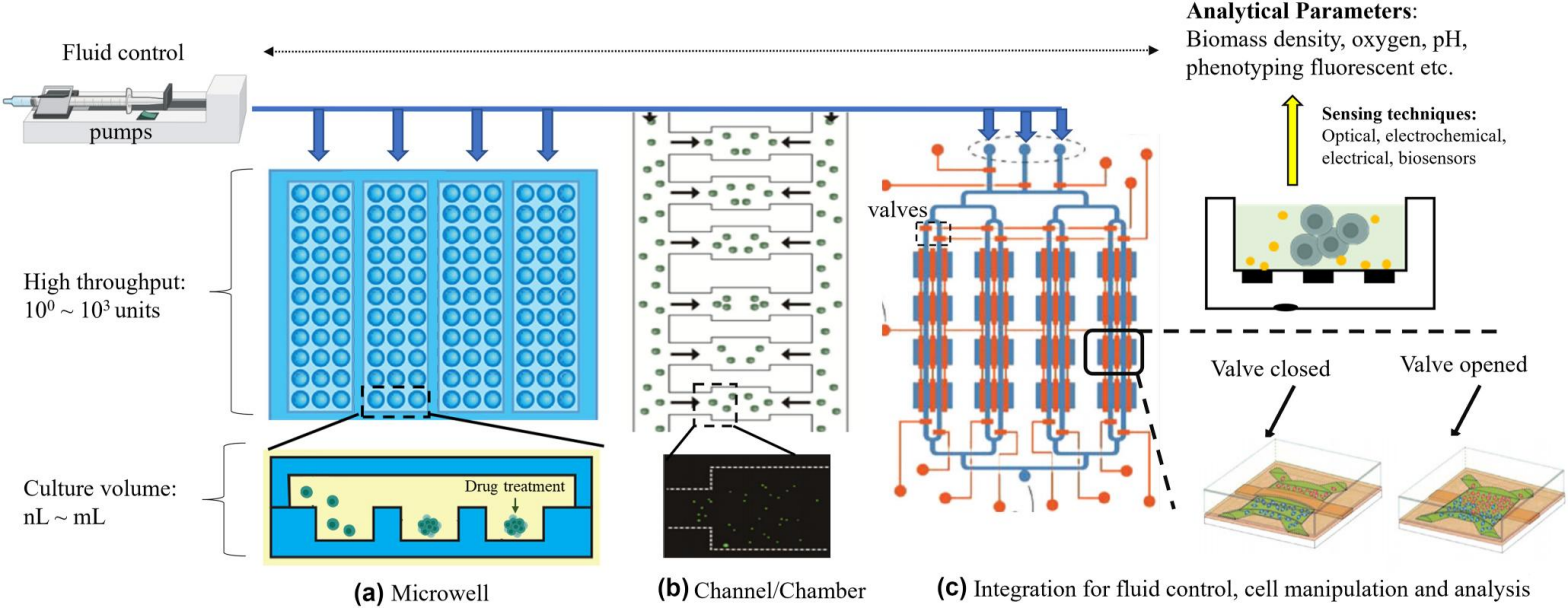
High‐throughput single‐phase flow microfluidic cell culture system. (a) Microwell‐based culture [70]. (b) Channel/chamber‐based culture [71], which are capable of adapting wide range of culture volumes (nL∼ml). (c) Multiplexing microfluidic platform with large integration (valves, pumps) for precise fluid control and cell manipulation [51]. Integration with several sensing techniques allows the real‐time acquisition of analytical parameters

A single‐phase microfluidic culture system refers to a dynamic cell culture process in millilitre or smaller scale microfabricated compartments and is usually incorporated with peripheral equipment to perform different automation. As shown in Figure 2, single‐phase culture devices can be classified into well‐based and channel/chamber‐based devices, and these devices can be further integrated with several culture platforms.

To ensure that certain devices serve as a promising cell culture platform, research has been focussed on the compartmentalisation for cell positioning, patterning, manipulation of diverse functions, and integrated sensing. With the advancements of microfabrication and configuration, automated cell seeding and patterning concepts have been proposed. The spatial separation and position of cells in the culture area are important for quantifying cellular dynamics [72, 73], cell heterogeneity study [44, 74], cancer cell biology [12, 75, 76] and single‐cell phenotypic analysis [71, 77]. Hydrodynamic cell capturing strategies used physical constraints incorporated with fluid flow to capture and isolate cells; these are common in many microfluidic devices, including the micro structured trap site on microwell arrays and side chambers along the main channel and physical micropillar barriers [71, 78, 79, 80]. A pneumatic‐controlled microstructure was integrated for precise localization and recovery of tumour cells [12]. In some studies, the chamber's bottom can be designed concavely to facilitate cell aggregation and spheroids formation [78, 81]. With the increasing demand of single‐cell analysis, to isolate a single cell from suspension, Lin et al. proposed a dual‐well concept in which the single cell is trapped in 25 μm diameter microwells and subsequently transferred into larger microwells by flipping the device upside down for further culture procedure [82]. Apart from the specifically structured chamber, addressable multiplexer and valve‐integrated device showed active fluid controllability, programmability in automated cell culture and patterned immunoassays [83]. Addressable valve‐integrated microfluidic devices have been used to perform extensive parallel cell culture and assays [84], for example cell‐micro Chip [1]. Additionally, direct access and recovery of cells in an enclosed chamber after culturing are crucial for further upscale culture or downstream DNA sequence analysis. In this regard, a pneumatic pump and solenoid valves‐integrated microfluidic prototyping platform capable of performing a non‐disruptive cell sampling process for ex‐situ analysis was presented. This proof‐of‐concept device suggested a ports‐connected plumbing system which offers accurate fluid delivery, targeted cell seeding and patterning, and further extended its application in 3D culture [50]. Another device featuring 1024 culture sites that enabled single cell trap, culture, selection and extraction was demonstrated to culture microalga *Chlamydomonas reinhardtii*. By opening the individual culture site that was controlled by the OR logic gate, backflow was selectively implemented in the culture region and thus targeted cells were extracted for downstream analysis [84]. On the other hand, a novel light‐actuated optofluidic technique has been integrated in the nano fluidic platform for higher accuracy of massive parallel single cell manipulation, cell selection, patterning, culturing, phenotypic analysis and selective extraction [85]. Mocciaro et al. used the Beacon® platform (Berkeley Lights, Inc) to conduct gene‐edited human primary T cell culture and fluorescent phenotypic assessment in NanoPens™ that consists of thousands of pens [86]. Certain level of precision and automation was achieved by the Opto‐electropositioning (OEP) technique that can generate light‐induced electrical gradient force in a photoconductive substrate and it was also applied for cell‐line development [87] and antibody detection [88, 89].

The single‐phase microfluidic system was primarily developed to perform continuous culture, and further adapted for various culture modes (e.g. long‐term culture, co‐culture and 3D culture). An integrated microfluidic chip with two‐positional injection valves was designed for long‐term culture of induced pluripotent stem cell (iPSCs). In this system, the exposure time of mouse embryonic stem cells to the GFP plasmid can be customised according to different flow conditions [10]. Moreover, programmable culture routines usually require feedback control on the automated operation (e.g. dilution of cell populations) with coupled key culture parameters (e.g. pH value, optical density, and dissolved oxygen). The dynamic control of culture conditions addressed the perturbation and made it possible to perform perfusotat, chemostat and turbidostat culture in a completely automated and controllable way, which can be further applied to genetic induction, evolutionary adaption study and optimization of synthetic genomes [19, 22, 27]. Additionally, a PDMS‐based microfluidic chip was used to facilitate the cell‐cell interaction study, such as tumour‐endothelial cell interactions [51], effects of adipocytes on insulin secretion dynamics [90], and pre‐symbiotic signalling dynamics between poplar‐associated fungi and bacteria [91]. More efforts have been made for the integration of three‐dimensional culture which is crucial for proper mammalian cell growth, modelling and migration [92]. For example, the multi‐well microfluidic platform was demonstrated to assemble the 3D cell culture with the cell microarrays that can be utilised for different applications for example drug screening, anti‐cancer drug activity analysis [93, 94] and long‐term tumour spheroid cultivation [95].

The ability of microfluidic system to achieve the dynamic monitoring and in situ analysis over the cell culture variables has gained popularity, because the valuable culture information, as well as the cellular responses, can be quantitatively measured for example direct coupling with mass spectrometry for real‐time cell analysis [11, 96], integrated electrochemical or biosensors for immunoassays [53], and non‐invasive impedimetric biomass density detection [26] as well as optical measurements of biomass [97], pH and oxygen via opdotes [98]. A multiparametric electrode integrated culture platform achieved real‐time monitoring of cellular metabolites of human brain cancer T98G cells, including pH, oxygen, lactate and glucose. The pH and oxygen sensors were placed at the inlet channel, within the culture chamber and outlet channel while the biosensor for metabolites production was integrated at the downstream culture process. In this situation, the metabolic rates of lactate secretion, extracellular acidification, cellular respiration activity and glucose uptake was also determined by applying the periodic feeding protocol [55]. Integrated culture‐based microfluidic systems with mass spectrometry technologies were used to facilitate semi quantitative cell metabolism studies [99], such as normoxia and hypoxia lactate concentrations of normal cells and cancer cells [100] and GH3 cells growth hormone secretion when co‐cultured with PC12 cells [96].

More recently, a study demonstrated functional secretory immunoassays in a valve‐integrated multiplexed microfluidic platform. Biosensors were immobilised in bio sensing modules while neutrophils and monocytes were cultured in adjacent chambers. After incubation in stimulating conditions, the mixing of secreted cytokines from the culture chamber with media in sensor modules was automated via valves actuation and secretion concentration was measured. The versatility of this device was also proved with assessments of IL‐8 secreting neutrophils and TNF‐α producing monocytes [56]. Another programmable valves‐integrated microfluidic device employed a microbead‐based in‐line electrochemical detecting strategy for hepatotoxicity assessment over 5 days [53]. The superior features and inherent advantages of integrated microfluidic cell culture systems have been widely used to perform cell research by providing reliable and reproducible cell culture performances. On the other hand, imaging from the bright field, phase contrast, time‐lapse live‐cell microscopies intuitively delivered detailed information of the dynamic division behaviour, the transformation of cells morphology and spontaneous‐induced stress behaviour [101]. It was attributed to the intrinsically higher spatiotemporal resolution of microfluidic technology.

In short, single‐phase flow microfluidic culture systems with integrated fluidic control technology, detection techniques and various microfabricated configurations have significantly contributed to the automation and streamline of culture process by realizing cell capture, multiple culture mode and monitoring at different resolution level.

### 2.2 Droplet‐based cell culture

**FIGURE 3:**
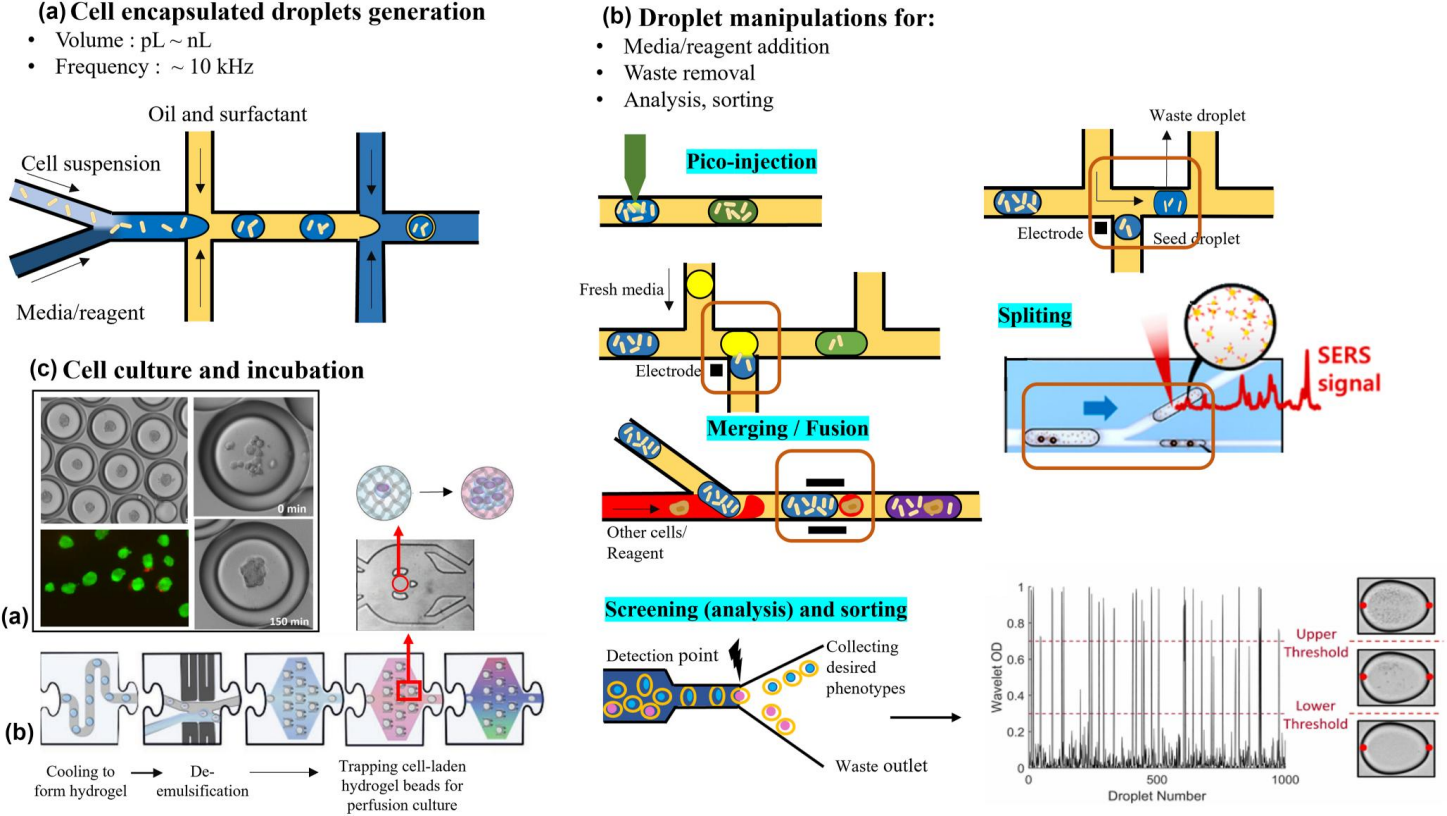
Integrated droplet‐based system culture process: (a) encapsulating cell in droplets, droplet sizes can be easily controlled by flow rate and channel dimensions. (b) Cell culture and incubation, for example (a) spheroids forming in *w*/*o*/*w* droplets [109], (b) long‐term perfusion culture single cell‐encapsulated hydrogel beads by trapping in microstructure sites [59]. (c) Integrated droplet manipulation strategies, such as pico‐injection, merging and fusion for reagent addition [110]; Splitting to remove waste droplets or sampling for analysis [111]; Screening and sorting droplets containing desired phenotypes [37]

Droplet‐based microfluidic system has been well‐recognized for its capabilities in high‐resolution cell compartmentalisation and multiplexed high‐throughput assays. Two or more immiscible phases (e.g. aqueous and oil‐phase, multi‐aqueous phase) are used to create spatially separated droplet compartments. The droplets with ultra‐low volume (typically pL – μL) serve as an isolated chamber and has been previously reported to be a promising culture platform for days [102]. In recent years, several cell encapsulated‐droplet forms including water‐in‐oil (*w*/*o*), water‐in‐oil‐in‐water (*w*/*o*/*w*) and multi‐aqueous droplets (*w/w*) generated in controllable frequency and uniform size have attracted great attention in quantitative biological applications including cellular heterogeneity drug screening [103], cell‐based antibody assay, dynamic cytokine secretion [104, 105], single‐cell phenotyping and genomics [106, 107], cell‐cell interaction studies [108], biomimetic 3D culture [109] and so on. Droplet microfluidics has emerged as a promising high‐throughput screening tool in biology analysis. Over these years, efforts have also been made to develop multiple droplets generation for cell compartmentalisation and manipulation strategies (e.g. transport, splitting, fusion, sorting and analysis) and automated culture operations on integrated platform, as demonstrated in Figure 3.

Cell‐encapsulated droplets’ generation strategies include flow‐induced shear stress [33, 45] and pump/valve actuation [48]. In most cases, hydrodynamical‐based shear stress techniques, for example flow‐focussing, co‐flowing and cross‐flowing in different microchannel configurations have been used to form cell‐encapsulated droplets at high frequency (for up to 10^3^ Hz) [32, 36]. Zhang et al. have successfully achieved single *Phaffia rhodozyma* cell culture in pL‐droplets for 80% success rate with over 90% of single‐cell isolation efficiency [107]. In addition, pump/valves provide an external mechanical force to actuate droplet formation [48]. These methods can generate a wide range of droplet volumes by simply modulating the flow rate, volume fraction and channel dimensions [112, 113], resulting in uniform single cells encapsulated in a single droplet. Recently, Qu et al. generated multicellular spheroids using the double emulsion (DE) technique and performed in situ characterisation by trapping spheroids in tailored microwells [114]. In this strategy, cells were spontaneously aggregated in DE droplets without the need of scaffolds. Moreover, Sun et al. generated core (tumour cells) – shell (fibroblast cells) hydrogel microcapsules for 3D co‐culture and drug screening [115]. Hydrogel capsules with hollow cores are known to help shaping cells into uniform spheroids [116]. The hydrogel droplets usually required several centrifugation and washing steps before the cross‐linking process (e.g. chemical chelator or ultraviolet light exposures) to tune their matrix properties before culturing [117]. Therefore, on‐chip integration of photo‐crosslinking and de‐emulsification functions boosted the operational efficiency of the whole hydrogel‐based culture process into next level of automation that leads to higher cell viability [45, 118]. Later on, cells‐laden hydrogel was trapped in a microchip, and subsequently used to perform long‐term perfusion culture [59] or cytokine secretion measurement [104]. Additionally, trapping and immobilising droplets in a microfabricated chamber array allow in situ analysis [119] and have increased the droplet's accessibility [120, 121]. A double‐layer microvalve‐integrated microfluidic device allowed selective manipulation (e.g. trapping, releasing and rearranging) on addressable pico‐litre droplets array that would be applied as the multiple biological analysis platform [122]. Certain static culture pattern also facilitates real‐time monitoring of the cell in indexed droplets [123].

It is important to perform several culture protocols (e.g. growth media refreshment, metabolites removal and reagent addition into droplets) for controllable growth dynamics [124], long‐term continuous culture [69, 125], adaptive evolution research [126] and cell‐based screening [42]for synthetic biology. These operations are generally executed in water‐in‐oil droplets for regulating the dynamic culture environment, which can be achieved through the interplay between microchannel geometries, microvalve and electrodes, such as pico‐injection, splitting and fusion [34, 42, 111, 125]. High precision pressure‐driven liquid control incorporated with valves and pico‐injection techniques have recognized droplet‐based chemostat microbial culture with biomass population variation <5% for over 50 h [125]. Merging, splitting, and mixing of droplets can be pre‐programmed and precisely executed, and therefore automated iterative liquid handling steps can be followed in adaptive evolution experiments. In this respect, cell‐incubated droplets can be split into waste and seed droplets by introducing electric forces, where seed droplets can be fused with fresh media (or chemical factors) and cultured media [34]. A fully automated microbial microdroplet culture (MMC) system was employed for selecting methanol‐dependent *Escherichia coli* mutants under minimal losses of the volatile substrate [126]. The utility of integrated multifunctional droplet microfluidic platform was demonstrated by Zhang et al. to resolve low system efficiency problem which was subjected to accumulation of errors, unstable droplets reflow in microchannel and limited throughput of bigger droplet manipulation. This system proposed an innovative strategy for the co‐cultivation study and drug screening which utilised reflowed droplets (encapsulated target cells) to physically cleave another aqueous flow (other cells or reagent) in a *Y*‐junction channel, and thus formed auto‐synchronized pairing droplets which can be later merged by electrocoalescence methods [110]. Combining this channel design with dielectrophoretic force, a droplet microfluidic device capable of cell washing and solution change is developed keeping cell loss less than 5%, significantly enhancing the droplet culture strategies and assay types [127].

Digital microfluidics (DMF) systems have been used as alternative liquid‐handling techniques to manipulate droplets (e.g. dispensation, splitting, merging, and mixing) in high precision and high fidelity manner by applying electrostatic forces on the substrate with integrated electrodes configuration instead of pumps, valves or channels [128, 129]. Ahmadi et al. combined DMF and droplet‐in‐channel microfluidic on the same device, that is the integrated droplet‐digital microfluidic (ID2M) system, to culture yeast mutants [130]. With the integration of electrodes, generation and manipulation of droplets eliminated the exquisite requirement of flow rate and timing control. Most interestingly, incubated cells in droplets can be accessed in a non‐serial manipulation manner and *n‐*ary sorting can be achieved (as opposed to typical binary sorting channels). The *n*‐ary sorting channels configuration enabled higher separation level of droplets that contained different concentrations of fluorescence to establish a potential high‐throughput screening platform for directed evolution experiments. In another study, Kumar et al. demonstrated the DMF system for analysis of single yeast cells [131]. This DMF platform was used in combination with the microwell array to trap the individual droplet and it allowed the high‐throughput cytotoxicity assays with improved spatio‐temporal resolution.

Additionally, the monitoring and sorting function of cell‐incubated droplets relied on non‐invasive optical‐based detection due to high sensitivity, robustness and ease of integration. Typically, high‐throughput droplet‐based screening strategies used optical measurements such as fluorescence intensity [14, 33, 35, 44, 132, 133] and optical density [34, 69] as quantitative signals according to the density of target molecules secreted or cells for the screening and sorting process [134]. Duarte et al. suggested a contactless conductivity detection strategy for real‐time measuring label‐free cell numbers in nL‐droplets with a detection limit of 63.66 CFU per droplet [135]. Hassanzadeh‐Barforoushi analysed single‐cell secretion in nL‐droplet systems [136]. This device utilised capillary methods to form and trap droplets in microchambers for real‐time fluorescent analysis of cell secretion dynamics. Choi et al. suggested a surface‐enhanced Raman scattering (SERS)‐integrated droplet culture system capable of the automated immunoassays procedure [111]. A versatile droplet chain array device capable of multimode cell manipulation (i.e. 2D/3D culture, co‐culture and migration) that adopted the porous PC membrane and droplets‐in‐hole‐array concept was demonstrated by Ma et al. [137]. This device can be further integrated with liquid handling systems for improved automation.

Briefly, droplet‐based microfluidics has demonstrated excellent culture and screening performance by implementing various manipulation strategies. However, more effort needs to resolve the technical issues for precise manipulation and automation of the whole‐cell culture process in ultra‐small volume droplets.

## 3 APPLICATIONS OF MICROFLUIDIC CELL CULTURE SYSTEM

**FIGURE 4:**
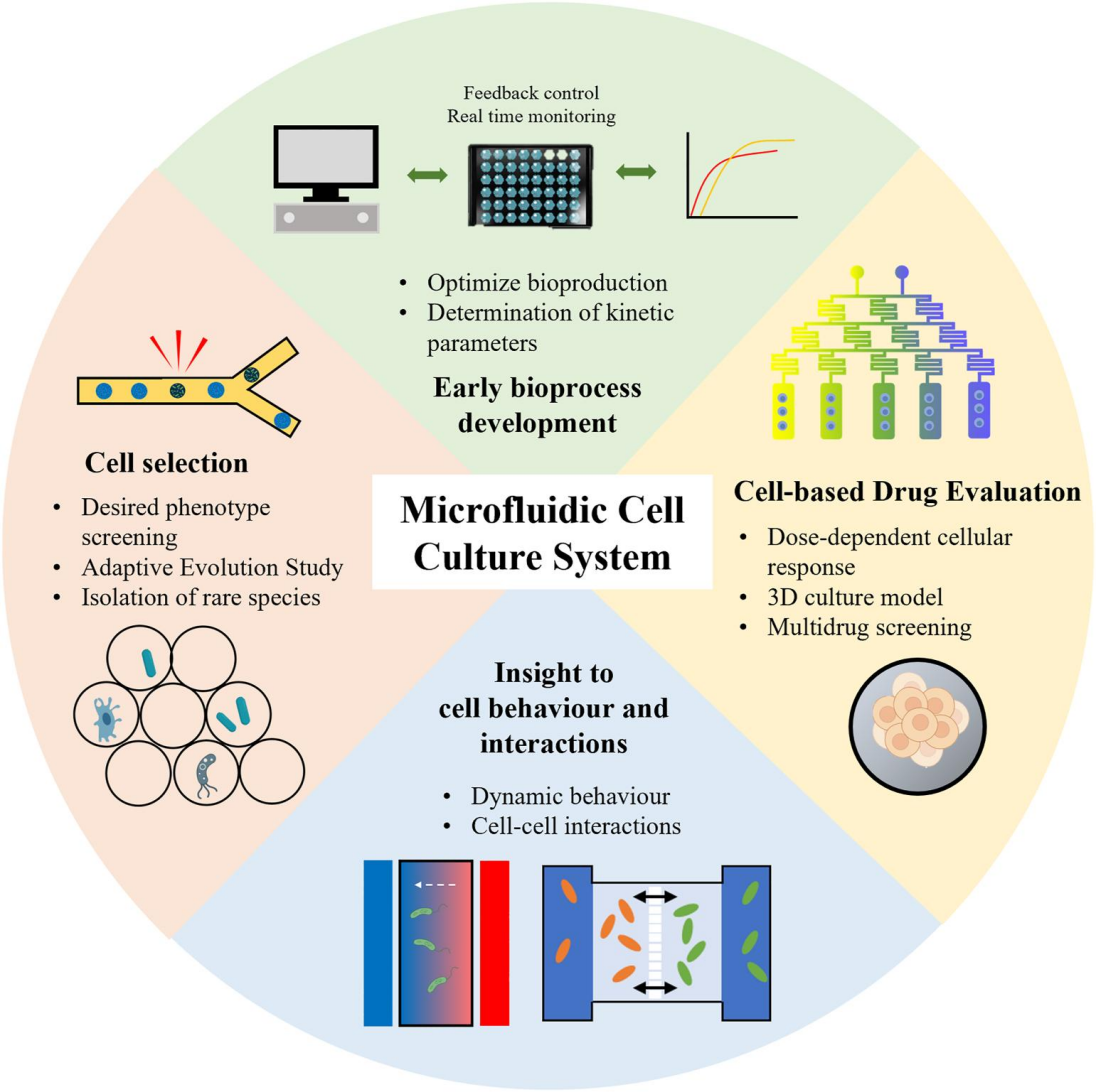
Applications of microfluidic cell culture systems in varieties of research fields

The superior feature and inherent advantages of integrated microfluidic cell culture systems which realised various culture modes have been widely applied (as summarised in Table 2), including early bioprocess development, cell selection, drug evaluation and deeper study of cell behaviour and interactions, as shown in Figure 4.

**TABLE 2:** Microfluidic applications for microbial and mammalian cells based on culture modes

Culture modes	Application	Cell types	Remarks
Batch	Screening for desired phenotypes	*Escherichia coli*	[138]
		*Saccharomyces cerevisiae*	[138]
		*Pichia pastoris*	[43]
		*Yarrowia lipolytica*	[42]
		Yeast	[32]
		*Aspergillus niger*	[33]
		Hybridoma cells	[44, 132]
	Antimicrobial susceptibility testing (AST)	*Escherichia coli*	[39, 40]
		*Klebsiella pneumoniae*	[40]
		*Staphylococcus aureus*	[40, 139]
		*Enterococcus faecalis*	[40]
	Cell migration	Human breast cancer cell lines, human neutrophils	[140]
	Cell‐cell interaction	*Clonostachys rosea, Fusarium graminearum*	[141]
	Determination of monod kinetic parameter	*Saccharomyces cerevisiae*	[24, 142]
Fed‐Batch	Multidrug screening	A549 cells	[143]
	Optimise feeding strategies	*Corynebacterium glutamicum*	[18]
Perfusion	Bioprocess development	Pichia pastoris	[27]
		*Saccharomyces cerevisiae*	[21]
		CHO cell line	[97, 144]
		C2C12, HFF cell lines and mouse embryonic stem cells	[145]
	Cell migration	Pseudomonas putida	[146]
		Human oral squamous cancer cell line, Human glioblastoma cell line	[147]
		Jurkat cell	[148]
	Cytotoxicity assay	HUVEC cells	[61]
Subculture	Adaptive evolution	*Escherichia coli*	[34, 126]
3D culture	Cytotoxicity assay	HeLa cells	[149]
		Colon cancer cells (HCT116)	[81]
	Cell‐cell interaction	Tumour‐endothelial cell	[118]

### 3.1 Early bioprocess development

Early bioprocess development usually requires abundant quantitative data associated to process variables to determine feeding strategy, media composition, secretion productivity and growth conditions. For this reason, the integrated, high‐throughput microfluidic culture systems enabled process control, reliable informative data acquisition and minimised time and labour cost. Recently, Totaro et al. characterised the lactic acid production of *Saccharomyces cerevisiae* strains in batch and perfusion culture modes by using a multifunctional microfluidic integrated with biosensors of DO and biomass concentration [21]. A fully automated and sensor integrated commercialised BioLector Pro microbioreactor system, micro‐Matrix system comprising several parallel culture wells was commercially used for effective process development tools, for example optimization of pH‐based feeding strategies of *Corynebacterium glutamicum* [18], and CHO cell perfusion culture [97, 144]. Additionally, continuous culture has gained increasing interests as it offers advantages of higher economic efficiency, lower power consumption and waste products. In this respect, a syringe pump‐controlled microfluidic device was developed to evaluate different media delivery schemes on human and murine cell lines (HFF and C2C12) long‐term perfusion culture [145]. Continuous and periodic feeding strategies that results in different spatiotemporal profiles of cell factors were investigated in terms of cell morphology, phenotype and homogeneity. Mozdzierz et al. employed a microfluidic system capable of closed‐loop feedback control over OD, DO and perfusion rates to evaluate protein production of hGH‐ and IFNα‐2b‐expressing *Pichia pastoris* strains [27]. Different methanol concentrations, perfusion rate and cell density were characterised to improve protein titres. Characterisation of dynamic cell growth and metabolites profile in response to fluctuations are valuable to determine the bioprocess conditions [2]. Microfluidic systems have also been used to determine kinetic parameters of *S. cerevisiae* under low substrate concentrations [24] and aerobic culture [142], where the reported kinetic values were comparable to larger‐scale bioreactor cultivation as published previously. More advanced microfluidic devices were also reported as a proof of concept to allow long‐term perfusion culture for mammalian cells and microbial cells [28, 150, 151], which can be potentially devoted for bioprocess development with further implementation of the programmed control system. The integrated microfluidic platform can provide the automation, high‐throughput experimental data, cost‐effective and time‐saving technology to meet the requirement of early bioprocess development.

### 3.2 High‐throughput cell selection

Due to the outstanding high‐throughput performance and detection efficiency, microfluidics becomes a promising screening approach for high‐yielded, robust and desired phenotype selection from engineered‐strain libraries or developed cell lines. Particularly, compartmentalised cells incubated in μL, nL or pL droplets allow rapid screening and sorting based on enzyme secretion [32, 42, 152], intra or extracellular compounds [133], antibodies [44, 132, 153] coupled with detectable signals as well as adaptive evolution [19]. Current techniques have allowed screening performance for up to millions of cells by several kHz [42, 102]. For example, mutant of filamentous fungi *Aspergillus niger* [33] and yeast strain [32] with improved amylase production, specific L‐lactate–producing *E. coli* strains as well as high xylose‐consuming *S. cerevisiae* [138] were isolated from 10^4^ to 10^5^ variants‐contained library using the electrode‐integrated device. With integrated droplets manipulation modules such as merging, fluorescent detection and sorting into one platform have broadened the versatility to accomplish the incubation task and measure metabolite production or consumption [138]. Besides that, functional alginate microbeads with encapsulated cells were used to capture specific binding antibodies for heterogeneous immunoassays [44]. By co‐encapsulating antibody‐secreting cells and cancer cells in millions *w/o* droplets, Shembekar et al. enriched specific binding antibodies by 220‐fold from 80,000 clones [132]. Jang et al. established an efficient screening droplet array platform combined with artificial riboswitch that responded to L‐tryptophan to quantify intracellular metabolite of *E. coli* [154]. On the other hand, an adaptive evolution experiment of methanol‐essential *E. coli* strains under gradually decreased gluconate pressure was conducted in the droplet‐based microfluidic system and 3‐fold improved growth rate with 40% gluconate consuming mutant was obtained after 50 days of cultivation [126]. This system allows subculture and adaptive evolution for improved characteristics of selected strains with precise droplet operations. Multiplexing eVOLVER system also demonstrated growth rate feedback control triggering fluid transfer to proceed with the automated yeast cells mating protocol [19]. The commercialised integrated microfluidic system that includes Cyto‐Mine® (Sphere Fluidic) is available to perform the cell isolation, selective screening and verification that could be applied in cell line development and antibody discovery.

Chambers or droplets confinement can facilitate the growth and enrichment of rare species and slow‐growing organisms which are often suppressed by fast‐grown species. This idea is mainly used for single‐cell analysis and genomics with in‐depth study of biodiversity associated with cell heterogeneity. Higher biodiversity and CFU concentration were reported in droplet‐based culture compared to petri dishes culture [36, 155], which contribute to the exploration of natural microbial communities. Watterson et al. successfully enriched rare taxa by stochastically encapsulating gut microbes in millions of droplets. In this experiment, 21 populations were revealed in the droplet‐based assessment for antibiotic resistance which were evaded in conventional plate culture [37]. Jiang et al. proposed a facile microfluidic streak plate method for accessible droplet array single‐cell culture for better coverage of rare species. Hundreds of isolates were obtained including undiscovered fluoranthene‐degrading Blastococcus species and demonstrated unravelled biodiversity of soil communities [29].

### 3.3 Cell‐based drug evaluation

Culturing cells under a predefined and reliable environment is crucial for dose‐dependent cellular response in drug screening events. Microfluidic devices comprising different multiplexing configurations proposed a high throughput, cost‐effective and functional manner to construct stable combinational drug gradients, and a 3D culture model with a reliable microenvironment (hypoxia, interstitial pressure). A typical application is to confine the cells in monodisperse droplets to increase cells’ density and substantially reduced time to reach the detection threshold [38, 39], whilst offering distinctive resolution to determine cell heterogeneity [156]. Antimicrobial susceptibility assessments of both Gram‐positive and negative bacteria including *E. coli, Klebsiella pneumoniae, Staphylococcus aureus, Enterococcus faecalis*, were conducted in ultra‐high‐throughput microdroplet docking array devices capable of four drug combinations simultaneously, where the test outcomes could be obtained within 30 min [40]. Multi‐drug screening has attracted attention in personalised antimicrobial and anticancer treatment with improved therapeutic efficacy [78, 143, 157, 158]. One study used a scalable multiplexed microfluidic chip to perform 172 drug combination assays on 1032 cancer spheroids simultaneously [78]. Lately, Lee et al. proposed a pneumatic micropumps and microvalves‐integrated device for on‐chip automation of bacteria dispensation, drug dilution and bacteria‐antibiotic mixing. The performance of this device was determined by assaying methicillin‐resistant *S. aureus* [139]. Another integrated droplet‐array microfluidic system capable of cell culture, scheduled drug dosing and cell viability assays was proposed to perform drug combination screening. These proposed multi‐drug screening microfluidic devices provide simple protocols and fast readout analysis, and thereby showed their great potential to develop as a clinical diagnostic tool in personalised therapy [143].

In cancer treatment, several studies have particularly concentrated on preserving the interaction between cells and the surrounding extracellular matrix to acquire more reliable dosing data. Colon cancer cells formed homogenous spheroids in concave microwells which subsequently perfused at five irinotecan concentrations in parallel [81]. Hydrogel‐based microdroplets have been used for 3D culture models due to their tunable physicochemical properties that can be mimicked in vivo extracellular matrix (ECM) nature. Wang et al. encapsulated HeLa cells in alginate‐matrigel mixed hydrogel beads for 4 days to culture uniform multicellular spheroids and suggested that tumour spheroid cells have higher vincristine drug resistance than conventional 2D monolayer culture [149]. On the other hand, the absence of oxygen control in conventional cell culture led to inefficient cancer therapy. In this case, an integrated microfluidic culture system was constructed to evaluate the hypoxia‐activated cytotoxicity of different anti‐drugs on A549 cells [16, 159], *Ishikawa* human endometrial cancer cell [160], and human breast cancer cell lines [161]. Moreover, multi‐fluid shear stress conditions were steadily established in one integrated microfluidic chip to investigate drug toxicity of HUEVC cells in the presence of fluid mechanical stimulations that mimicked interstitial and lymphatic flow in vivo [61]. These models are very important for obtaining more reliable cellular responses in an authentic microenvironment. Commercialised clinical microfluidic devices include ClearBridge and Vortex chips for label‐free detection and isolation of circulating tumour cells (CTCs) [162]. The clinical diagnostic device produced by Creative Biolabs is available for automated sample pre‐treatment, processing, reaction and also for pathogen and cancer detection. The 3D cell culture chips (AIM BIOTECH) were also manufactured for cancer therapeutics studies [163]such as T cell efficiency, metastasis and cell migration. In short, attributing to enhanced throughput, sensitivity, dosing accuracy, testing efficiency and accurately engineered microenvironment of drug evaluation process, integrated microfluidic devices have been devoted to a more reliable and effective diagnostic point‐of‐care system or the development of personalised therapy. More informative reviews about the microfluidic system for cell‐based drug screening have been published recently [156, 164].

### 3.4 Insight to cell physiology and interactions

Culturing cells at single‐cell and population levels has provided the opportunity to gain deeper insight into single‐cell behaviour, metabolism mechanisms [165] cell‐cell interactions [141], cell signalling pathway [96], cancer cell metastasis and cell migration. The capability to define the manifestation of cell behaviour through a high spatiotemporal microenvironment is essential to investigate the interplay mechanism between the cells. For example, a recent study focussed on filamentous fungal interactions in confined and segmented channels to quantitatively evaluate hyphal growth. This microfluidic platform overcomes the extensive hyphal branching on conventional plate culture and provides a new way to track hyphal by using live‐cell imaging [141]. Moreover, Burmeister et al. reported a microfluidic device with adjacent microchambers and separated nanochannels barrier structure that was used to investigate synthetic commensalistic co‐culture of two *C. glutamicum* strains as well as contact‐based gene transfer interaction between *E. coli* and *Pseudomonas putida* [30]. These small chambers combined with time‐lapse imaging provide the full spatiotemporal observation of the cellular response with single‐cell resolution. It also helps to understand the single‐cell activity without being masked with bulk cellular behaviours. A detailed study for microbial co‐culture focussed on physiology, growth dynamics and cellular interaction has been reviewed lately [166]. Co‐culturing cells proposed a relatively complete cellular environment to preserve natural function, regulating extracellular metabolites secretion or even cell signalling and stress response. For example, hepatocyte function was significantly enhanced in terms of increased albumin secretion and cytochrome P450 3A4 activity for 24 days co‐culture with endothelial progenitor cells [167]. An integrated microfluidic device comprising three functional modules (co‐culture, protein detection, drug metabolites pre‐treatment) was used to facilitate the investigation of the interaction between cervical carcinoma tumour cells and human umbilical vein endothelial cells. Higher chemo resistance and cell viability with increased production of angiogenic proteins and paclitaxel metabolites resulted in a co‐culture manner [168].

Besides that, microbial behaviours and cellular dynamic processes are valuable information to reveal the underlying physical and molecular mechanisms. The chemotactic response appeared as a dynamic process for cell migration towards or away from chemical stimulus for critical analysis of immune response [140, 169], tumour cell invasion [147], and targeted pollutants [146]. Microfluidic platforms proposed the better spatiotemporal gradient generating strategies that are advantageous for chemotaxis analysis of various cell types under chemically and physically defined culture conditions [66]. Shear‐free or flow‐free gradient conditions can be formed in microfluidic systems to study cell behaviour that is sensitive to fluid flow [148, 169]. More quantitative analysis can be obtained through the characterisation of essential parameters, for example chemotactic receptor constant and sensitivity coefficient [146]. Different advanced technologies such as optical bright‐field and high‐resolution time‐lapse imaging have been widely used in microfluidic systems for intuitive interpretation, quantitative analysis and real‐time monitoring on cell morphology, cell trajectories at cellular and molecular resolution.

## 4 CONCLUSION AND FUTURE PROSPECTS

Over the years, integrated microfluidic cell culture systems have revolutionised cell biology, biotechnology and biomedical disciplines by offering many advantages to meet the demands of high throughput, automation, multifunction integration, cost‐effectiveness, minimal reagent consumption and better spatiotemporal control over the microenvironment. Microfluidic devices have demonstrated equivalent or even better culture performance compared to that of conventional methods. In microfluidic cell culture systems, the implementation of manipulation function and sensing techniques automates overall workflow (cell culture, manipulation, and analysis) in a programmable, controlled manner and thus contributed to increase the throughput, parallelisation and efficiency of biological processes. The present review aims to unfold the superiorities of integrated microfluidic platforms in cell culture‐related processes that have been utilised in bioprocess development, drug screening and biological related fields. We also presented the latest applications of microfluidic culture systems for various cell types in many fields with advancements in multi‐functional performance and integration techniques. In conclusion, the previous research to date has proven the unprecedented flexibility of integrated microfluidic devices to develop as versatile cell culture systems.

As we can easily conclude that the multifunctionality and automation of microfluidic systems come up with the higher demand of instruments (robustness, compatibility, and sensitivity), the complexity of system configuration (multiplexing, delicacy) and technical requirements. These characteristics also led towards increased standardisation of the overall process as well as commercialisation. However, there are still many technical barriers for the extensive commercialisation and universal applications of integrated microfluidics systems due to the high demand of variable criteria. For example, in single‐phase microfluidics platforms, the culture conditions can be easily controlled with spatial coordinates. However, the ability of indirect cell manipulation (such as quantitative sampling) by a fluid‐controlled manner within enclosed microchamber is still limited. A large integration will be required for programmable cell manipulation in certain devices, and this would lead to an increase of cost. In contrast, it will be still difficult to encode the culture parameters of each droplet. Therefore, the metabolic changes in terms of oxygen intake, pH or nutrients consumption need to be encoded by fluorescent probes, which would have potentially brought negative effects on cell growth in such small volumes. The integration of droplet manipulation strategies for multifunction and fully automated culture processes remained a challenge due to the high demand of flow control under nL or smaller scale. For example, the manipulation of water‐in‐oil droplets will require high precision manipulation techniques to regulate the distances between two adjacent droplets [110]. Besides that, droplet handling steps for downstream analysis in an oil‐mediated droplet‐based system to remove oil and recover cells and metabolites can be tedious [118]. The droplet‐to‐digital (D2D) platform which allows individual droplets extraction for off‐chip downstream analysis can overcome this limitation [170]. Additionally, to circumvent unstable flowing droplets in microchannel and ensure the metabolites’ encapsulation efficiency, there are limited applications for oil‐soluble molecules and viscous substances‐secreting cells. Effective surfactants have to be appropriately applied to resolve droplets crosstalk issues that are caused by diffusible hydrophobic molecules (e.g. fluorophores) [123]. In this respect, the novel all‐aqueous‐phase droplets have reported higher cell viability due to elimination of the organic solvent, but low interfacial tension between two aqueous phases has hampered the stability and eventually led to relatively low throughput compared to that of water/oil systems [46, 47]. Moreover, the fluorescence signal has been the most general real‐time detection method to quantify labelled molecules or cells in the microfluidic platform, however, it requires the gene modifications or staining process which could lead to the undesired decrease of cell viability. Rapid label‐free cell‐based analytical methodologies such as the electrical impedance technique, dielectrophoresis measurements, and acoustic manipulation have consequently emerged to be potentially integrated for cost‐effective, accurate cell‐based assessments [171, 172, 173].

Despite the cost‐effective materials applied in microfluidic platforms for the cost‐saving purpose, in fact the sophisticated instrumentations, such as integrated sensors and valves as well as complicated set‐ups for establishing a microfluidic system requires highly specialised personnel to handle it, which restricted their generalized applications in laboratories. Meanwhile, troubleshooting, valid performance, determining variability and reproducibility of microfluidic systems require standard fabrication of microfluidic chips and operation protocols. For example, assembling various tubing and microfluidic chip requires exquisite manipulation or degassing process to avoid introducing air bubbles into it [23, 174]. Therefore, the commercialisation of integrated microfluidic platform offers standardized and automated protocols to realize lab on a chip. Indeed, microfluidic technology has been successfully commercialised for the past decades, for example Fluidigm Corporation provides the microfluidic platform for single cell genomics, viral detection and immunology applications. Other companies such as Micronit Microfluidics and Dolomite Centre offer microfluidic functionalities such as chip manufacturing, flow control, sensor integration and so on. Most of these successful companies provide full service of microfluidics products development and solutions, that is a ready‐to‐use microfluidic system with fixed functions such as single cell analysis, immunoassay diagnostics and genomic sequencing. Additionally, their modular microfluidic components can be customised and assembled according to variable customer's needs. It is clear to see that standardized protocols have greatly contributed to the commercialisation of microfluidic systems.

The major directions for further development of microfluidic systems will highlight the improvement in automation, standardisation and multi‐functionality of operational protocols as well as availability of the basic technologies for untrained staff in certain fields. The automation and standardisation of integrated microfluidic platforms with exclusive software for programmable protocols can increase the reproducibility of culture performance and facilitate technology translation or even transition to commercialised mass production [175, 176]. The plug‐and‐play concept incorporated with versatile interfaces can be employed to adapt different microfluidic chip designs and also couple with different downstream analysis methodologies in one system. Standard modular operation of the microfluidic system could achieve higher experimental flexibility and functionality [177] by assembling different functional modules with plugs like Lego® bricks [178]. To implement this idea, a standard and robust interconnection and inter‐compatibility is required between modules and systems [175] and it would be appropriate to fulfil the requirements of various types of cell culture and manifold analysis, such as reconstruction of organ‐on‐chips according to specific needs [176]. Although utility and potential of microfluidic systems in cell‐related studies are significant, but the combination of different scientific disciplines such as fluidics, mechanics, electronics, automation, chemistry, biology and engineering are expected to further improve its application and commercialisation.

Attributing to the intrinsic features of high throughput and better spatial‐temporal resolution, innovative microfluidic cell culture platforms have been devoted to ascending research in different areas of microbiology, biomedical and biotechnology. Typically, this emerging technology is capable to deal with the complexity of natural microbial living niches in a higher experimental resolution by considering the heterogeneity of the single or sub colony microbial cells that greatly contributed to cell biology and biodiversity study. Hence, we envision this advanced technology will exploit our understanding towards the microbial community as certain important studies are limited [179] by performing phenotypic screening and gene sequencing of cell encapsulated droplets, integrated channels are helpful to distinguish between systemic and localized effects which have already proven to be valuable to study the interactions of bacteria, fungus and nematode [180, 181]. We believe that the construction of microecosystem on microfluidic platform will also become another future research trend to solve the challenging problems related to microbial growth, functional phenotypes discovery, metabolic regulation and realizing the dynamic analysis, response and directional evolution of cells under different environmental stimulation conditions. On the other hand, the future endeavours of microfluidic technology will also focus on combination of advanced engineering tools with computer technology and big data information processing, making full use of its capability for large‐scale parallel culture, parameter detection and collection. In the case of precision medicine, customized therapeutic strategy and dosage administration could be determined through microfluidic diagnostic devices [182, 183]. The integrated microfluidic devices‐enabled sequential process (e.g. cell seeding, incubation and automated analysis) is envisioned to develop as an effective clinical diagnostic point‐of‐care device to meet the requirements of lower sample consumption, higher efficiency and detection sensitivity for clinical tests. Recently, chip‐mass spectrometry (Chip‐MS) has been used for clinical cell‐based analysis, it can also facilitate the studies of microbial metabolism, treatment of diseases and drug screening to characterize the wide range of molecules from different metabolism activities [100, 184]. Although lots of microfluidic devices have been developed, there are major hurdles in the microfluidic design for cell analysis in a more biomimetic microenvironment since the minor difference in microenvironments of cells can influence their phenotypes. We expected that a more multi‐functional integration of microfluidic devices will be gradually developed and applied in the clinical field in near future. In sum, the awareness and availability of microfluidics technology will be increased in near future with collaborations of multiple disciplines to make it a truly generalized and more reliable technology in scientific, industrial and clinical applications.

## CONFLICT OF INTEREST STATEMENT

No conflicts of interest.

## PERMISSION TO REPRODUCE MATERIALS FROM OTHER SOURCES

None.

## Data Availability

No data available.
